# Psychopathological Burden and Quality of Life in Substance Users During the COVID-19 Lockdown Period in Italy

**DOI:** 10.3389/fpsyt.2020.572245

**Published:** 2020-09-03

**Authors:** Giovanni Martinotti, Maria Chiara Alessi, Chiara Di Natale, Antonella Sociali, Franca Ceci, Lorenza Lucidi, Elena Picutti, Francesco Di Carlo, Mariangela Corbo, Federica Vellante, Federica Fiori, Gaia Tourjansky, Gabriella Catalano, Maria Luisa Carenti, Chiara Concetta Incerti, Luigi Bartoletti, Stefano Barlati, Vincenzo Maria Romeo, Valeria Verrastro, Fabio De Giorgio, Alessandro Valchera, Gianna Sepede, Pietro Casella, Mauro Pettorruso, Massimo di Giannantonio

**Affiliations:** ^1^Department of Neuroscience, Imaging, Clinical Sciences, University G.d’Annunzio of Chieti-Pescara, Chieti, Italy; ^2^Department of Clinical and Pharmaceutical Sciences, University of Hertfordshire, Herts, United Kingdom; ^3^Department of Mental Health, ASL Lanciano-Vasto-Chieti, Chieti, Italy; ^4^Pathological Addictions Treatment Division, La Promessa o.n.l.u.s., Rome, Italy; ^5^Department of Mental Health and Addiction Services, ASL RM1, Rome, Italy; ^6^Neurology and Neurorehabilitation Unit, Santa Lucia Foundation (IRCCS), Rome, Italy; ^7^Department of Mental Health and Addiction Services, ASL Alessandria, Alessandria, Italy; ^8^Department of Mental Health and Addiction Services, ASST Spedali Civili, Brescia, Italy; ^9^Department of Clinical and Experimental Sciences, University of Brescia, Brescia, Italy; ^10^Faculty of Psychological Sciences and Techniques, Dante Alighieri University, Reggio Calabria, Italy; ^11^Department of Medical and Surgical Sciences, University “Magna Graecia” of Catanzaro, Catanzaro, Italy; ^12^Division of Legal Medicine, Institute of Public Health, Catholic University of Sacred Hearth, Rome, Italy; ^13^Pathological Addictions Service, Villa S. Giuseppe Hospital, Hermanas Hospitalarias, Ascoli Piceno, Italy

**Keywords:** substance use disorder, addiction, COVID-19, craving, psychopathology

## Abstract

**Background:**

Following the development of the COVID-19 pandemic, a rigid public health strategy of reduced social contact and shelter-in-place has been adopted by the Italian Government to reduce the spread of the virus. In this paper, we aim at evaluating the impact that the COVID-19 pandemic, and the relative containment measures, have had on a real-life sample of patients suffering from substance use disorders (SUDs) and/or behavioral addictions.

**Methods:**

An anonymous questionnaire was filled out by 153 addicted patients, both outpatients and residential inpatients, recruited across Italy and highly representative of the current Italian population suffering from addictions. Psychopathological burden (anxiety and depressive symptomatology, somatization, irritability, and post-traumatic symptoms), quality of life, and craving changes in daily habits were assessed.

**Results:**

In our sample, we found moderate rates of depression (22.9%), anxiety (30.1%), irritability (31.6%), and post-traumatic stress (5.4%) symptoms. Psychopathological burden was globally higher among residential patients. Reported levels of craving were generally low.

**Discussion:**

This study is the first attempt to collect Italian data regarding the effects of the rigid quarantine period, during the COVID-19 pandemic, on patients suffering from a SUD and/or behavioral addictions. The presence of a moderate psychopathological burden correlated to poor quality of life and low craving scores represented the main outcomes. Long-term studies, with follow-up after the end of the restrictive measures, should be considered to implement our findings.

## Introduction

Following the advent of the COVID-19 pandemic, which developed between March 11, 2020 and May 3, 2020, social containment measures were implemented across Italy through a series of consecutive ministerial decrees aimed at limiting the spreading of the virus. The lockdown soon proved effective for such purposes, but at the same time, it generated an important series of consequences from both a social and an economic point of view. Social distancing, emotional isolation, complete transformation of the daily routine, abrupt adoption of an unhealthy lifestyle (sedentary lifestyle and unbalanced nutrition), and economic difficulties resulting from the interruption of work activities have thus compromised, and could continue to do so, the well-being of each individual and the entire community (1). Within the general population, problems such as feelings of frustration, aggressive behavior (2), post-traumatic stress symptoms (PTSS), depression, anxiety, insomnia, perceived stress, and adjustment disorder symptoms (ADS) have increased (3), with the consequent risk of self-medication through the abuse of alcohol and/or psychoactive substances and with a greater tendency to engage in pathological behaviors (gambling and internet addiction). It is possible that, among patients with pre-existing mental disorders, the symptomatology may flare up or worsen (with important management difficulties for the caregivers); the risk to develop suicidal ideation is also plausible for the most critical cases (1, 4, 5). The aforementioned effects in terms of mental health can be superimposed on those observed during other major epidemics/pandemics that have occurred in former times. Ebola (6), Human H7N9 Avian Flu (7), Middle East Respiratory Syndrome (MERS) (8, 9), and severe acute respiratory syndrome (SARS) (10–13) have in fact caused a real “mental health catastrophe” (12) among the affected population, above all amid the frontline workers managing the health emergency and among those who have recovered from the infection, including their relatives.

In this context, people with pathological dependencies on psychoactive substances and/or with behavioral addictions are particularly vulnerable. There is a real “collision” between SUDs and the COVID-19 infection. Moreover, drug users exposed to social risk factors, such as belonging to under-privileged social classes or, even worse, being homeless or imprisoned, are more often subject to precarious hygiene and health conditions. They are particularly susceptible to contract the infection, and, by virtue of obstructive and cardiovascular comorbidities of the ischemic- hypertensive type, they are prone to develop the disease in its most serious forms (14, 15). In patients with alcohol use disorder, the effects of the lockdown are notpredictable: social isolation, restricted freedom, and the resulting difficulties in obtaining the substance could lead to a reduction in the dysfunctional behavior. Nonetheless, an increase in withdrawal symptoms, and the possible use of DIY alcohol products, might have significant health fallouts and, potentially, even lead to death (16, 17). Among active users, a scarce availability of drugs, hence a reduction in their usage, could lead to withdrawal symptoms that are difficult to manage at home (5). Patients who are recovering from substance use experience psychological discomfort from social isolation, which might increase the risk of relapse. This alarming scenario is exacerbated by a quantitative and qualitative reduction in the addiction services’ assistance and in the stretching of their services (18): For instance, recovering patients’ access to support groups is prevented, and other forms of psychosocial assistance are limited as well (14). The handling of the substitution therapies for opiates addiction, in particular methadone and buprenorphine, has proven to be particularly complex, with difficulties in both supplying and distributing the aforementioned drugs (5, 14, 16, 17). These critical issues, caused by the rigid regulations that still guide the provision of replacement treatments, are similar to those documented in the past, e.g., following the terrorist attacks of September 11, 2001 on the Twin Towers and following Hurricane Katrina and Hurricane Sandy that hit the United States, respectively, in 2005 and 2012 (19). This implies a greater tendency to resort to illicit trafficking of opiates whenever the replacement drug cannot be found and increases the risk of death from possible overdose of the replacement drug, every so often dispensed to the patient in doses that are suitable to cover a greater period of time (17). Therefore, it is evident that the COVID-19 health emergency crisis collides with another important public health emergency, which is that of SUDs (14).

The aim of this study was to evaluate the impact that the COVID-19 pandemic, and the relative containment measures adopted by the Italian Government, had on patients with SUDs and/or behavioral addictions; to assess the psychopathological burden in terms of depression, anxiety, post-traumatic load; and to evaluate the relevance of craving symptoms and their correlation with psychiatric symptoms and quality of life.

## Materials and Methods

### Participants and Procedure

From March 11, 2020 to May 3, 2020, throughout the whole Italian lockdown phase, we carried out a survey meant only for adult people with an ongoing and/or previous SUD and/or gambling.

Disorder (DSM-5) currently in treatment as outpatients and/or in a residency program as inpatients. Two hundred twenty-seven patients were recruited and offered the possibility to fill out the questionnaire. One hundred fifty-three patients gave their consent and completed the questionnaire. The survey was conducted in two ways: through a self-administered paper questionnaire and through an online platform where the subjects filled out the questionnaire independently using an URL (uniform resource locator) provided by the clinician during an interview. The survey was completed by each subject anonymously only after having read the information sheet and having signed the informed consent form. Various centers for recruitment were randomly selected in different regions of Italy (Abruzzi, Calabria, Lazio, Piedmont, Marche, Lombardy, and Molise) in order to guarantee an equal distribution of the sample’s population around the country. In each recruitment center, a psychiatrist gave the survey to all eligible subjects. The presence of a DSM-5 diagnosis of SUD had been assessed and confirmed before the study procedures, representing an inclusion criterion of the study.

### Survey *S*tructure and Measurements

The survey was organized in three sections.

In the first section, we collected anamnestic information and clinical variables that included age, gender, education level, relationship status, days spent in lockdown, primary substance of abuse, substitute and/or support treatments, pathological gambling, support by addictions services, comorbid psychiatric disorders and psychopharmacological treatment, hospitalization, and SARS-Cov-2 testing. In the second section, we asked the subjects to indicate the level of craving for the primary substance of abuse and how much their craving and habits have changed since the start of lockdown. We used a visual analogue scale (VAS), which ranged from 1 (strongly reduced) to 10 (strongly increased). We investigated the change in quality of life, the consumption of cigarettes, coffee, alcoholic drinks, cannabis, cocaine, opioids, benzodiazepines, food, and the time spent shopping online, instant messaging, and making video calls with friends/relatives on social networks, carrying out old and/or new hobbies, in sport activities, watching TV series or films, and watching pornographic material. In the third section, we investigated the psychopathological variables of interest, from the start of the lockdown to the completion of the survey. Irritability was measured using four irritability items from the Irritability depression anxiety scale (IDAS) (20); five items from the self-rating anxiety state (SAS) were employed to investigate anxiety (21). Somatic symptoms were investigated with a single question about the presence of all possible pathological conditions. The Davidson trauma scale (DTS) was adopted for the assessment of post-traumatic stress symptoms (22), and the beckdepression inventory - II (BDI-II) (23) was utilized to assess current depressive symptoms. According to the scores obtained in the scales, symptomatology was divided into two categories: minimal/mild and moderate/severe.

### Statistical Analysis

Statistical analysis was performed using Statistica 8.0 (Statsoft Inc. Usa, 2007). Quantitative parameters were presented as mean ± standard deviation (SD) and qualitative parameters as number and percentage per class. Kolmogorov-Smirnov (K-S test) was used to check for the normality of distributions. Analysis of variance (ANOVA) and Duncan *post hoc* test were utilized to evaluate the differences among subgroups’ means. The associations between variables were measured using Pearson’s correlation. The p value was considered significant if <0.05.

## Results

### Sample Characteristics

Most patients were males (n = 119, 77.8%); the mean age was 39.8 (± 12.3) years. At the time of questionnaire completion, the subjects had been in quarantine for an average of 47.3 (± 14.1) days. Most subjects (n = 66, 43.1%) indicated cocaine as the principal substance of abuse, followed by alcohol (n = 39, 25.5%) and THC (n = 24, 15.7%). Of the entire sample, 97 patients (63.4%) were outpatients, living at home during quarantine, while 56 (36.6%) were inpatients in residential programs. The full participants’ characteristics and the substances’ patterns of use are presented in **Table 1**.

**Table 1:** Participant’s characteristics and pattern of substance use.

	Mean	SD
**Age**	39.8	12.3
	**N**	**%**
**Gender**		
Male	119	77.8
Female	34	22.2
**Education level**		
None	1	0.7
Primary school	8	5.2
Lower secondary school	36	23.5
High school	82	53.6
Bachelor's degree/Postgraduate degree	24	15.7
**Relationship status**		
Single	66	43.1
Widow / widower	1	0.7
Divorced	22	14.4
In a relationship	51	33.3
In a relationship but not seeing each other till the beginning of the lockdown	11	7.2
**Having children**		
Yes	33	21.6
No	95	62.1
	**Mean**	**SD**
**Days spent in lockdown**	47.3	14.1
	**N**	**%**
**Quarantine violations**		
Yes	26	17.0
No	123	80.4
Not in quarantine	3	2.0
**Sars-Cov2 testing**		
Negative	33	21.6
Positive	3	2.0
None	116	75.8
Data unavailable	1	0.7
**Cigarette smoking**		
No	32	20.9
Yes occasionally	12	7.8
Yes < 10 cigarettes/day	24	15.7
Yes 10-20 cigarettes/day	57	37.3
Yes > 20 cigarettes/day	27	17.6
**Primary substance/behavior of abuse**		
Cocaine	66	43.1
Alcohol	39	25.5
THC	24	15.7
Gambling	12	7.8
Heroin	9	5.9
Benzodiazepines	1	0.7
Ketamine	1	0.7
Psychopharmacological medications	1	0.7
**Secondary substance/behavior of abuse**		
Cocaine	12	7.8
Alcohol	14	9.2
THC	17	11.1
Gambling	20	13.1
Opioids	4	2.6
MDMA / Meth-amphetamines	3	2.0
Nicotine	1	0.7
Not specified	4	2.6
None	78	51.0
**Gambling**		
Yes	33	21.6
No	109	71.2
In the past	11	7.2
**Days from the last use of the primary substance**	95.6	81.6
	**N**	**%**
**Followed by an Addiction Service**		
Yes	93	60.8
No	60	39.2
**Substitute and/or support treatments**		
Methadone	11	7.2
Buprenorphine	3	2.0
Naloxone/naltrexone	4	2.6
Sodium oxybate	8	5.2
Disulfiram	1	0.7
**Hospitalization**		
Inpatients	56	36.6
Outpatients	97	63.4

THC: Δ9-tetrahydrocannabinol; MDMA: methylenedioxymethamphetamine.

Sixty-seven (43.8%) participants reported a comorbid psychiatric condition, especially mood disorders (depression and bipolar disorder) or anxiety. Sixty-three (94%) of those with comorbid psychiatric condition and 26 (30.2%) of those without a comorbid psychiatric disorder reported undergoing psychopharmacological treatment. All the information regarding the comorbid psychiatric conditions and their pharmacological treatments remained unchanged. About 10% of the patients reported a comorbid medical condition. Only one subject (0.7%) had a COVID-19 related pneumonia (**Table 2**).

**Table 2:** Psychiatric comorbidity in the full sample.

	N	%
**NO PSYCHIATRIC COMORBIDITY**	**86**	**56.2**
No psychopharmacological treatment	60	69.8
Monotherapy	16	18.6
Polytherapy	10	11.6
Antidepressants	13	15.1
Benzodiazepines	11	12.8
Antipsychotics	3	3.5
Mood stabilizers	13	15.1
**WITH PSYCHIATRIC COMORBIDITY**	**67**	**43.8**
Depressive disorder	18	26.9
Bipolar disorder	14	20.9
Anxiety disorder	11	16.4
Borderline personality disorder	5	7.5
Psychotic disorder	5	7.5
Cyclothymic disorder	2	3.0
Obsessive compulsive disorder	2	3.0
Attention deficit hyperactivity disorder	2	3.0
Paranoid personality disorder	1	1.5
Eating disorder	1	1.5
Unspecified	6	9.0
Psychopharmacological treatment		
No therapy	4	6.0
Monotherapy	17	25.4
Polytherapy	46	68.6
Antidepressants	43	64.2
Benzodiazepines	38	56.7
Antipsychotics	18	26.9
Mood stabilizers	45	67.2

### Psychopathology, Quality of Life, Craving

We calculated the total score for five psychometric scales (IDAS-irritability, DTS, SAS-five items, somatization, and BDI-II) in both the entire sample and in five of the principal categories of substances/behaviors (alcohol, cocaine, gambling, THC, and heroin). ANOVA showed no significant effect on the principal substance of abuse (**Table 3**).

**Table 3:** Results of the psychometric scales and substances/behaviors, ANOVA results.

	ValidN	IDAS -irritability		DTS		SAS -five items		SOM		BDI-II	
		Mean	SD	Mean	SD	Mean	SD	Mean	SD	Mean	SD
Alcohol	39	7.9	3.1	22.0	23.4	9.5	3.8	1.7	1.0	12.7	11.5
Cocaine	66	7.5	2.7	22.2	22.5	9.5	3.4	1.7	0.9	12.0	11.4
Gambling	12	5.7	1.2	22.3	23.0	9.1	2.5	1.3	0.5	11.8	9.8
THC	24	6.7	2.3	22.7	17.7	9.3	3.1	1.7	1.0	11.6	8.7
Heroin	9	6.8	2.4	23.8	16.5	9.6	3.0	1.8	0.7	20.3	14.8
Total	153	7.4	2.7	22.1	21.4	9.4	3.3	1.7	0.9	12.7	11.3
	df	F	p	F	P	F	p	F	p	F	p
ANOVA	4;145	2.08	0.09	0.01	0.99	0.05	0.99	0.73	0.57	1.20	0.31

THC, Δ9-tetrahydrocannabinol; BDI-II, Beck depression inventory–II; SAS–five items, Five items from the self-rating anxiety statee; DTS, Davidson trauma scale; IDAS-irritability, four irritability items from the irritability depression anxiety scale; SOM, somatization.

Each psychopathological domain was scored into two levels of severity: minimal/mild and moderate/severe. Scores are detailed in **Table 4**.

**Table 4:** Results of the psychometric scales and ranges (cases and %).

**BDI-II**	
*Minimal/mild (0–19)*	*Moderate-severe (20–63)*
118 (77.1%)	35 (22.9%)
**SAS-five items**	
*Minimal/mild (1–10)*	*Moderate-severe (11–20)*
107 (69.9%)	46 (30.1%)
**DTS**	
*Minimal/mild (0–67)*	*Moderate-severe (68–136)*
140 (94.6%)	8 (5.4%)
**IDAS-irritability**	
*Minimal/mild (1–8)*	*Moderate-severe (9–16)*
104 (68.4%)	48 (31.6%)
**SOM**	
*Minimal/mild (1–2)*	*Moderate-severe (3–4)*
125 (82.2%)	28 (17.8%)

BDI-II, Beck depression inventory–II; SAS–five items, five items from the self-rating anxiety state; DTS, Davidson trauma scale; IDAS-irritability, four irritability items from the irritability depression anxiety scale; SOM, somatization.

The mean level of craving was generally low (3.4), nonetheless a general low difficulty in finding the substances of abuse was reported. The level of craving was higher in outpatients (mean = 3.8) compared to inpatients (mean = 2.8, p = 0.038) (**Table 5**).

**Table 5:** Craving visual analogue scale (VAS) in different subgroups, ANOVA results.

	N	Mean	SD
CRAVING VAS IN FULL SAMPLE	153	3.4	3
CRAVING VAS IN INPATIENTS	56	2.8	2.8
CRAVING VAS IN OUTPATIENTS	97	3.8	3.1
ANOVA results: F (1; 151) = 4.36, p < 0.05. Duncan post hoc test: Outpatient > Inpatient			

VAS, visual analogue scale.

The association between the level of craving for the principal substance of abuse and the values of the psychometric scales was measured using Pearson’s correlation. These data about craving will be further elaborated elsewhere. The level of significance (p = 0.05) was corrected for multiple comparisons using the Bonferroni correction: p corr = 0.05/n comparisons = 0.01. We observed a significant positive correlation between the level of craving and the mean total values of DTS, SAS, (five items) and BDI-II, and the results remained significant after Bonferroni correction (**Table 6**).

**Table 6:** Pearson correlations between craving and psychometric results. * = significant after Bonferroni correction (p corrected = 0.01).

	R	P
IDAS-irritability	0.01	0.99
DTS	0.24	0.003*
SAS-five items	0.22	0.006*
SOM	0.14	0.09
BDI-II	0.34	0.0001*

BDI-II, Beck depression inventory–II; SAS–five items, five items from the self-rating anxiety state; DTS, Davidson trauma scale; IDAS-irritability, four irritability items from the irritability depression anxiety scale; SOM, somatization.

When comparing inpatients versus outpatients by means of ANOVA, the IDAS (irritability) scale resulted in significantly higher levels among inpatients. Comparing dual diagnosis participants against non-dual diagnosis participants, BDI-II, DTS, and somatization scores were significantly higher among dual-diagnosis patients. VAS quality of life scored higher in the non-dual diagnosis group. Results of ANOVA tests are detailed in **Table 7**.

**Table 7:** ANOVA results comparing dual diagnosis and non-dual diagnosis participants.

Psychometric scale	Dual-diagnosis subsample	Non dual-diagnosis subsample	F	p
IDAS-irritability(mean ± SD)	7.7 ± 2.8	7.2 ± 2.8	1.12	0.292
DTS (mean ± SD)	30.1 ± 24.5	16.4 ± 16.6	16.67	0.000
BDI-II (mean ± SD)	17.2 ± 12.8	9.4 ± 8.7	19.62	0.000
SAS-five items(mean ± SD)	10.1 ± 3.9	9.1 ± 2.7	3.623	0.059
SOM (mean ± SD)	1.9 ± 1	1.5 ± 0.9	5	0.027
VAS quality of life(mean ± SD)	3.9 ± 2.3	4.8 ± 2.5	5.09	0.026

BDI-II, Beck depression inventory–II; SAS-five items, five items from the self-rating anxiety state; DTS, Davidson trauma scale; IDAS-irritability, four irritability items from the irritability depression anxiety scale; SOM, somatization; VAS, visual analogue scale.

We found an increase of about 50% of the cases for the amount of time spent on the following daily activities: eating, instant messaging, social networking, video calls to friends/relatives, watching movies/TV shows, and sleeping. About 40% of subjects increased their online search to gather information about the ongoing pandemic.

## Discussion

This study collects the first Italian data regarding patients suffering from SUDs and/or behavioral addictions during the rigid quarantine period caused by the COVID-19 pandemic. The study, which includes patients recruited in seven different representative Italian regions, has the uniqueness of incorporating previously treated patients who were known by local services and who were all given a DSM-5 diagnosis of SUD. In addition, the recruited group represents a real-life sample that reflects the Italian addiction scenario (24) and was homogeneously differentiated into residential and non-residential patients, with some patients reporting a dual diagnosis and others none.

The psychopathological burden observed in our sample is in line with recent international data concerning psychiatric patients, subjects with dual diagnosis, and drug addicts. The effects of quarantine on mental health have been highlighted in a recent review that evaluates the psychological distress among the quarantined people during past pandemics and epidemics (25). Many studies, based on online surveys, have shown an increase in anxiety, depression, and stress among Chinese (26–29), Italian (3, 30), and Spanish (31) people due to the COVID-19 pandemic. Our results are in line with these findings, showing relatively high rates of depression, anxiety, irritability, and post-traumatic stress symptoms among the sample. Specifically, 22.9% of our sample reported moderate/severe depressive symptoms, and 30.1% reported moderate/severe anxiety symptoms, similar to what was indicated by another Italian survey that rated 32.8% of participants as having high/very high depressive symptoms and 18.7% of them as having high/very high anxiety symptoms (30). These results show no substantial psychopathological difference between our sample and the general population. Mazza et al. reported a considerable increase in the use of telephones, social networks, and mobile apps toconnect with family and friends during the quarantine period among the Italian population. Our findings are in line with these results, showing an increase in the use of instant messaging (51.6%) and video calls (54.9%) to connect with friends and relatives among substance users as well. Moreover, we found an increase in the time spent utilizing social networks (47.7%), collecting online information about the current situation (40.5%), and watching movies or TV shows (60.1%). In our study, the level of craving resulted to be overall, lower than real-life samples of Italian patients with SUDs (32). Craving is one of the key symptoms in addicted patients, closely correlated with the prognosis and progression of the pathology (33) and lower levels could influence positively the treatment outcome (34). This unexpected result could be explained by a perceived lack of availability of the substance that interrupted the development of the craving priming and by the presence of decreased social pressure on a group of subjects that are usually excluded and stigmatized. Specific craving variations between the lockdown-period and prior times will be reported and discussed elsewhere. Craving was higher among outpatients than inpatients. This data underlines the importance of residential treatment in SUDs. In fact, numerous studies demonstrate the effectiveness of this approach in increasing the perceived quality of life and in improving executive functions and psychological distress (4, 14, 19), conditions that lead to a reduction in craving (35). Such a notion is relevant because substance craving is a known predictor of relapse after treatment for SUDs (36). Residential treatment could, therefore, be a fundamental first step in laying the foundations for subsequent long-term outpatient treatment. This is even more true if we take into account that it also causes a change in the perception that the drug addict has of himself, transitioning from a ‘substance user’ social identity to an ‘in-recovery’ identity (37). In terms of craving intensity, the benefits of the presence of strict limitations on personal freedom, including the impediment to obtain substances, combined with the benefits of carrying out intensive treatment in residential structures, are perhaps the most interesting result of our study and it has relevant therapeutic implications.

Moreover, our results underline the link between craving and quality of life, defined as the perception that the individual has regarding the effects that a disease, and its treatment, have on his physical, emotional, and social well-being (38). More than half of the cohort reported reduced quality of life during COVID-19 lockdown, and the analysis showed a negative correlation between perceived quality of life and reported craving. The association between alcohol craving and quality of life was previously studied by Herrold et al. in war veterans demonstrating that high levels of craving were associated with poor perceived quality of life, bothmentally and physically (39). At the same time, improving the quality of life, for instance, through physical exercise, can play an important role in reducing craving and, therefore, conducts of abuse (40). Several studies have demonstrated that stress, negative mood, and craving could expose addicted patients to relapse and dropout from treatment (41). These factors are important elements of vulnerability that can be correlated with each other. It is essential to recognize and treat each one of them to improve the outcome. In fact, in our analysis we found a positive correlation between craving and depressive symptoms, anxiety, and traumatic stress. These findings are in line with the study of Fatseas et al. that found an association between psychiatric distress, mood and/or anxiety disorders, and higher levels of craving (42). Moreover, Luminet et al. found strong correlations between negative affect and craving in alcohol-dependent patients. In their study, an increase in depressive symptoms was related to increased levels of craving in women (43). It is necessary to look for the association between craving and psychopathological conditions because it could present useful information for a successful treatment. Specific attention to these clinical parameters could be the basis for a specific strategy to be employed in those populations exposed to the pandemic and to its associated restrictions and could open new scenarios based on possible preventive interventions. In lockdown period, the role of telepsychiatry acquires great importance for careful monitoring of the patient’s clinical and psychopathological conditions in order to prevent relapses (44). Through telematic interview, the clinician can also supervise the patient’s family environment, trying to understand if it provides the patient with enough support.

This study has some limitations: 1) the absence of a long-term follow-up, potentially useful to highlight the consequences of the lockdown; 2) in a part of the sample, the survey was completed online directly by the patient without proper verification by the clinician; and 3) the assessment of craving, which has always been complex and sometimes difficult to interpret, was carried out with a visual analogue scaling and not with more structured scales.

Long-term studies, with follow-up at the end of the restrictive measures and after the full development of the psychopathological experience caused by the pandemic and by its socio-economic consequences, may clarify the true impact of the COVID-19 pandemic on those subjects affected by SUDs. Meanwhile, thanks to this study being conducted with a sample of Italian drug addicts, it was possible to identify a moderate psychopathological burden correlated with poor quality of life and craving scores. The latter were overall low, especially among patients who are hospitalized in residential structures, opening interesting questions in terms of treatment strategies.

## Data Availability Statement

The raw data supporting the conclusions of this article will be made available by the authors, without undue reservation.

## Ethics Statement

Ethical review and approval was not required for the study on human participants in accordance with the local legislation and institutional requirements. The patients/participants provided their written informed consent to participate in this study.

## Author Contributions

GM, MA, AS, and CN contributed to conception and design of the study. MC, GT, GC, MLC, CI, LB, SB, VR, VV, FG, AV, and PC contributed to the recruitment of the sample and the organization of the article’s sections. MP, MA, and FDC organized the database. GS and FDC performed the statistical analysis. MA, AS, CN, FC, LL, EP, FF, and FV wrote sections of the manuscript. MP, GM, and MG performed the critical revision and approved the article. All authors contributed to the article and approved the submitted version.

## Conflict of Interest

GM has been a consultant and/or a speaker and/or has received research grants from Angelini, Doc Generici, Janssen, Lundbeck, Otsuka, and Pfizer. MG has been a consultant and/or a speaker and/or has received research grants from Angelini, Janssen, Lundbeck, Otsuka, Pfizer, and Recordati.

The remaining authors declare that the research was conducted in the absence of any commercial or financial relationships that could be construed as a potential conflict of interest.

The reviewer, JC, declared a shared affiliation, though no collaboration, with one of the authors, GM, to the handling editor.
